# In the diffuse large B-cell lymphoma microenvironment, autophagy genes are upregulated in pro-inflammatory macrophages and linked to *BCL2* overexpression

**DOI:** 10.3389/fimmu.2025.1676563

**Published:** 2025-12-03

**Authors:** Miguel Resanoa, Alba Delgado, Claudia Rodiño, Marina Villar, Ana Aiastui, Mounia S. Braza

**Affiliations:** 1 Department of Surgical Pathology, University Hospital of Navarra, Navarra, Pamplona, Spain; 2 Biogipuzkoa Health Research Institute, San Sebastian, Spain; 3 Department of Oncological Sciences, Icahn School of Medicine at Mount Sinai, New York, NY, United States; 4 Ikerbasque Basque Foundation for Science, Bilbao, Spain

**Keywords:** lymphoma, macrophages, inflammation, autophagy, BCL2

## Abstract

Diffuse large B-cell lymphoma (DLBCL) is one of the most frequent B-cell non-Hodgkin lymphoma types. It is characterized by a complex immune microenvironment, rich in macrophages (innate immunity cells), and high aggressiveness. DLBCL cells might respond to the increased energy demand by enhancing key metabolic processes, such as autophagy in which damaged cell constituents and debris are sequestered/removed for recycling. Here, we investigated the autophagy gene expression profile in DLBCL and in non-tumor controls using publicly available gene expression datasets and a substantial cohort of patients’ tissue samples. For the first time, we describe in the DLBCL microenvironment, a differential autophagy gene expression profile characterized by overexpression of *BCL2* (anti-apoptotic factor) in M1 pro-inflammatory macrophages compared with M2 immuno-suppressive macrophages. Moreover, the expression levels of *CD86* (M1 macrophage marker) and *CSF1R* (M2 macrophage marker) were positively correlated with those of *BECN1* (autophagy regulator) and *BCL2* (only *CD86*) that were in turn correlated with *MTOR* expression in tumor B cells and in the *CD86^+^* macrophage subtype. We confirmed these results by immunohistochemistry and immunofluorescence analyses of DLBCL and non-tumor tissue samples. Our finding of an autophagy-related pro-inflammatory signature highlights the crucial role of autophagy in the DLBCL immune microenvironment and suggests its potential as a therapeutic target.

## Introduction

Most lymphoid tissue malignancies (90%) are classified as non-Hodgkin lymphomas (NHL). This group of blood cancers is characterized by a complex and deregulated immunological response (1) due to specific tumor-supportive cells that become corrupted/biased when interacting with tumor B cells (2, 3). Diffuse large B-cell lymphoma (DLBCL) is one of the most common NHL types. It is characterized by a highly proliferative behavior and morphological heterogeneity with a diffuse architecture: most tumor lymph nodes do not show a regular morphology because of the infiltration by medium-to-large B cells with huge nucleoli and cytoplasm. These features explain the variable clinical course and response to therapy (1, 4–7). Moreover, this aggressive and very active cancer has an increased energy demand and therefore, its metabolism deregulation (for instance, through autophagy and apoptosis alterations) could be targeted by new therapeutics (8, 9).

Autophagy is a key metabolic process in which unnecessary or dysfunctional cellular components are removed through degradation/recycling (10–13). Autophagy can have tumor suppressive or tumor supportive roles, depending on the cancer origin and type, and the tumor microenvironment composition (13–16). Beclin-1 (encoded by the *BECN1* gene) is a key autophagy factor involved in the initiation of this process (17). However, when deregulated, it could play a tumorigenic role (18). Moreover, beclin-1 physiologically interacts with Bcl-2 (encoded by the *BCL2* gene), an important anti-apoptotic protein that is overexpressed and often mutated in follicular lymphoma and DLBCL (19). Their interaction regulates autophagy by determining the fate (autophagy or apoptosis) of the concerned cells (17).

We previously showed that the DLBCL immune microenvironment is characterized by a strong infiltration of pro-inflammatory M1 macrophages that promote the inflammatory state in this lymphoma (20). We also found that SIRT1 (a metabolic function regulator implicated in autophagy) is overexpressed in DLBCL and its expression level is correlated with the infiltration of pro-inflammatory M1 macrophages and linked to a pro-autophagic signature (under revision). Therefore, in the present study, we wanted to determine the autophagy and apoptosis gene expression profiles in DLBCL, particularly in M1 macrophages. To this aim, we exploited publicly available gene expression data on 48 DLBCL (TCGA database) and 337 control (GTEx database) samples. Then, we validated these results in independent DLBCL (n=128) and control (n=20) samples included in different tissue microarrays (TMA). Overall, we found that in the DLBCL microenvironment, key autophagy and apoptosis factors were upregulated. However, by comparing their expression profiles in the M1 and M2 macrophage subtypes, *BECN1* and *BCL2* were overexpressed only in pro-inflammatory M1 macrophages and correlated with mTOR expression.

## Materials and methods

### Computational biology datasets

To perform the bioinformatics analysis, we used a TCGA dataset of 48 DLBCL tumor samples (patients’ characteristics in **Supplementary Table S1**, **Supplementary Figure S1**) and a GTEx dataset of 337 control samples.

### Tissue microarray samples

For the immunohistochemistry (IHC) and immunofluorescence (IF) analyses, we used several commercially available TMAs: TMA1 (TissueArray, LY2086b) that includes 176 lymphoma samples of different subtypes (118 DLBCL, 3 Burkitt-like lymphoma, 5 follicular lymphoma, 1 mantle cell lymphoma, 4 plasma cell lymphoma, 7 anaplastic large cell lymphoma, 22 T-cell lymphoma, 4 angioimmunoblastic T-cell lymphoma, 12 Hodgkin’s lymphoma samples) and 16 lymph node samples as controls; TMA2 (TissueArray, MC1081) that includes 108 samples of different blood cancer types (20 leukemia, 10 DLBCL, 10 other non-Hodgkin’s lymphoma, 20 Hodgkin’s lymphoma, 11 plasma cell myeloma, 9 extramedullary plasmacytoma, 20 malignant thymoma samples), and 8 normal lymphoid organs as controls (2 lymph node, 2 spleen, 2 bone marrow and 2 thymus gland tissue samples); and TMA3 (TissueArray, CTRL141) that includes 14 controls (2 lymph node, 2 spleen, 2 bone marrow, 2 tonsil, 2 placenta, 2 appendix, and 2 thymus gland tissue samples, in duplicate). From these TMAs, we analyzed only 128 DLBCL and 38 control samples to validate the *in silico* findings at the protein level. Among the control samples, we used 20 non-tumor lymph nodes as negative controls and the other lymphoid organs (e.g. spleen, bone marrow) as positive controls to validate the IHC and IF staining protocols.

The patients’ characteristics are summarized in **Supplementary Tables S2** (DLBCL samples) and S3 (controls).

### Gene expression profiling interactive analysis

As previously described (20), we used the GEPIA server (21, 22) and its new version (23) and publicly available mRNA sequencing data for differential gene expression profiling in non-tumor control (GTEx dataset) and DLBCL (TCGA dataset) samples. Using the CIBERSORT-ABS, EPIC and quanTIseq tools, we performed a deconvolution analysis of each sample tool in TCGA/GTEx. For each bulk RNA sample, starting from the cell proportions, we performed downstream analyses, such as proportion, correlation, sub-expression and survival. We used boxplots to display the results of the gene expression and deconvolution analyses, and dot plots to summarize the results of the correlation analyses.

### CBioPortal for cancer genomics

We used the CBioPortal server (https://www.cbioportal.org/) to transform the multimodal cancer genomic data of the TCGA DLBCL cohort into interactive graphs and to summarize the patients’ main characteristics, as previously described (24–26).

### TIMER2.0

As previously described (27–29), we used the TIMER2.0 server (http://timer.cistrome.org/) and different algorithms, such as CIBERSORT-ABS and EPIC, for the deconvolution analysis of cell type and subtype signatures to evaluate the tumor-infiltrating immune cell types. We used the list of differentially expressed genes in DLBCL *vs* non-tumor samples to identify changes in tumor-infiltrating immune cell populations.

### Immunohistochemistry procedure

For IHC, we used DLBCL and control samples in commercially available TMAs. After heat-induced epitope retrieval, we incubated sections (4 °C, overnight) with primary antibodies against CD86 (Novus Biologicals, AF141, USA), CSF1-R (Abcam, Ab183316, UK), beclin-1 (Abcam, ab11407, UK), Bcl-2 (Abcam, Ab182858, UK) and mTOR (Abcam, ab109268, UK), followed by incubation with biotin-streptavidin horseradish peroxidase-conjugated secondary antibodies and 3,3′ -diaminobenzidine. We scanned each slide with a Philips Pathology Scanner SG300 and analyzed the obtained images with the Philips IntelliSite Pathology Solution image management system. We used the IHCExpert artificial intelligence tool to quantify the percentage of positive cells per core. In addition, a pathologist (co-author in this manuscript) validated all the quantitative results using a light microscope (Olympus BX41, model U-DO). We evaluated the histoscore of each sample as previously described (20).

### Immunofluorescence procedure

For IF staining, we used DLBCL and control samples in the same commercially available TMAs. After drying the TMA sections at 73°C for 10 minutes followed by dewaxing and rehydration using decreasing concentrations of alcohol, we performed antigen recovery by incubation in Tris-EDTA buffer, pH 9, in a microwave for 25 minutes. Then, after non-specific binding inhibition by incubation in 10% donkey serum for 1 hour, we incubated sections (4°C, overnight) with primary antibodies against CD20 (ab64088, Abcam, UK), CD68 (ab201340, ab213363, ab289671, Abcam, UK), CD86 and CSF1-R (AF141, NBP1-43362, Novus, UK), Bcl-2 (ab692, Abcam, UK), and mTOR (ab109268, Abcam, UK). This was followed by incubation with the secondary antibodies at room temperature for 1 hour and then with Sudan Black for 1 hour to reduce autofluorescence. We acquired images at 20X and 40X magnification with a Zeiss AxioObserver 7 microscope and ZEN, version 3.7. We quantified and qualified cell staining manually in triplicate in each TMA core (DLBCL and controls).

For IF data interpretation, particularly when specific antibodies could not be combined or used, we determined the peri-tumor (peri-T) and/or intra-tumor (intra-T) localization of the cells and their characteristics (size and morphology) to correlate the IF signal to the main cell population (macrophages and tumor cells). A pathologist (co-author in this manuscript) validated the analysis and results.

### Statistical analysis

For quantitative data, we used fold changes, ranks and correlation coefficients. We considered significant p-values ≤ 0.05. For the TCGA and GTEx datasets, we evaluated the significance of the gene expression correlation analyses by computing the Pearson, Spearman and Kendall correlation coefficient values and P-values. In GEPIA, we used the non-log scale for calculation and the log-scale axis for visualization. As shown in the boxplots, we compared differential gene expression data, cell type proportions and sub-expression analyses between DLBCL and non-tumor control samples using one-way ANOVA. We used the Mantel–Cox test to estimate the survival contribution of specific autophagy- and inflammation-related genes expressed in DLBCL and displayed them as log10 hazard ratios (HR). We displayed the IHC and IF quantitative results as percentages ± SD and compared them with the two-tailed unpaired Student’s *t*-test.

## Results

### The expression levels of many autophagy and apoptosis factors are increased in the DLBCL microenvironment and are correlated with *BCL2* and *BECN1* expression

**Figure 1:**
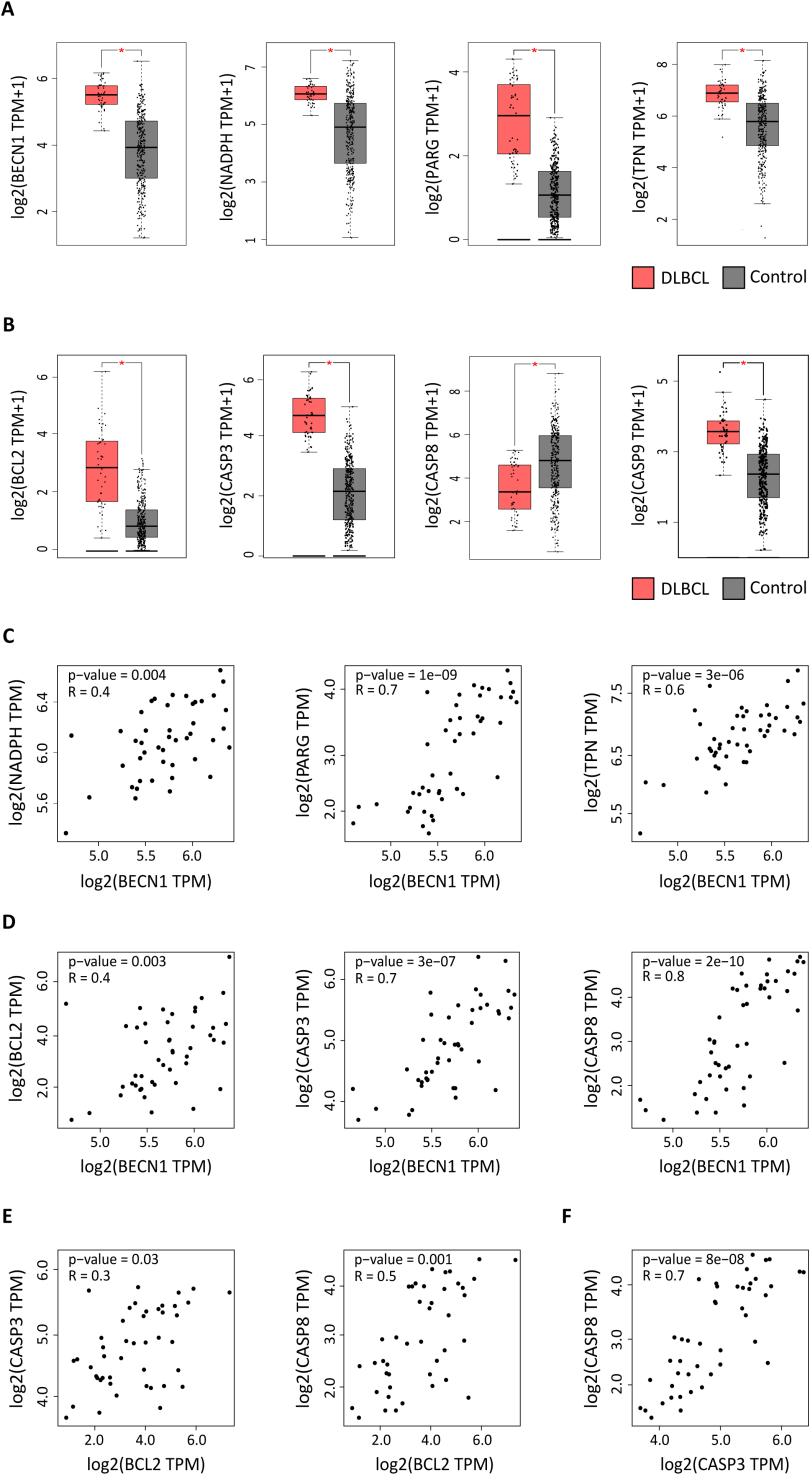
Expression of autophagy and apoptosis factors in DLBCL and control samples. Expression profiling of autophagy and apoptosis genes in 47 DLBCL (TCGA database) (red boxes) and 337 non-tumor control (GTEx database) (gray boxes) tissue samples. **(A)** Expression levels of the *BECN1*, *NADPH*, *PARG*, and *TPN* autophagy factors (log2 (TPM + 1). **(B)** Expression levels of the *BCL2*, *CASP3*, *CASP8*, and *CASP9* apoptosis components (log2 (TPM + 1). TPM: transcript count per million reads. **(C)** Correlation of *BECN1* expression with *NADPH*, *PARG* and *TPN* expression levels. **(D)** Correlation of *BECN1* expression with *BCL2*, *CASP3* and *CASP8* expression levels. **(E)** Correlation of *BCL2* expression with *CASP3* and *CASP8* expression levels. **(F)** Correlation of *CASP3* and *CASP8* expression levels. *p ≤ 0.05 (one-way ANOVA).

In our previous study, we identified a high pro-inflammatory signature in the macrophage-rich DLBCL microenvironment (20). Moreover, we found that SIRT1 and SIRT3, two sirtuins with key roles in metabolism regulation, are upregulated in the macrophage-rich DLBCL microenvironment and that SIRT1 expression is correlated with autophagy in M1 pro-inflammatory macrophages (under revision). Now, in the last part of this project, we asked whether autophagy is deregulated in M1 and M2 macrophages from the DLBCL microenvironment and whether the autophagy gene expression signatures are different in these macrophage subpopulations. To this aim, first, we compared the gene expression profiles of 48 DLBCL samples (TCGA database) and 337 healthy control samples (GTEx database). *BECN1, NADPH, PARG* and *TPN* were significantly upregulated in DLBCL samples compared with controls (p ≤ 0.05) (**Figure 1A**). Moreover, comparison of their expression in several cancer types (from the TCGA database) showed that *BECN1* (the key autophagy factor) was specifically overexpressed in DLBCL samples (**Supplementary Figure S2A**). Therefore, we selected *BECN1* as reference autophagy factor for the next analyses. Then, as the *BCL2* anti-apoptosis factor is frequently overexpressed in NHL and specifically in DLBCL (30), we evaluated its expression profile in several cancer types (from the TCGA database) and we confirmed its overexpression specifically in DLBCL and acute myeloid leukemia (AML) (**Supplementary Figure S2B**). Therefore, we compared the expression profiles of the main apoptotic factors in the 48 DLBCL samples from the TCGA database and 337 healthy control samples. We found that *BCL2, CASP3, CASP9* were significantly upregulated and *CASP8* was significantly downregulated in the DLBCL samples compared with controls (p ≤ 0.05) (**Figure 1B**).

Next, we focused on *BECN1* and *BCL2* and asked whether their upregulation in DLBCL was correlated with the expression levels of other autophagy (*NADPH*, *PARG*, *TPN*) and apoptosis (*CASP3*, *CASP8* and *CASP9*) components. *BECN1* expression level was positively correlated with that of *NADPH*, *PARG* and *TPN* (autophagy factors) (r = 0.4, p =0.004; r = 0.7, p = 1e-09 and r = 0.6, p = 3e-05) (**Figure 1C**), and of *BCL2, CASP3* and *CASP8* (apoptosis components) (r = 0.4, p =0.003; r = 0.7, p = 3e-07 and r = 0.8, p = 2e-10) (**Figure 1D**). Similarly, *BCL2* expression level was positively correlated with that of *CASP3* and *CASP8* (r = 0.3, p = 0.03; r = 0.5, p = 0.001) (**Figure 1E**). Moreover, *CASP3* and *CASP8* expression levels were positively correlated (r = 0.7, p = 8e-08) (**Figure 1F**).

Altogether, these results suggest that in the DLBCL microenvironment, some autophagy and apoptosis genes are upregulated and that *BECN1* and *BCL2* expression levels are correlated with those of other autophagy and apoptosis factors.

### In DLBCL, the expression of autophagy and apoptosis components is correlated with the expression levels of the macrophage markers *CD68*, *CD86* and *CSF1R*

**Figure 2:**
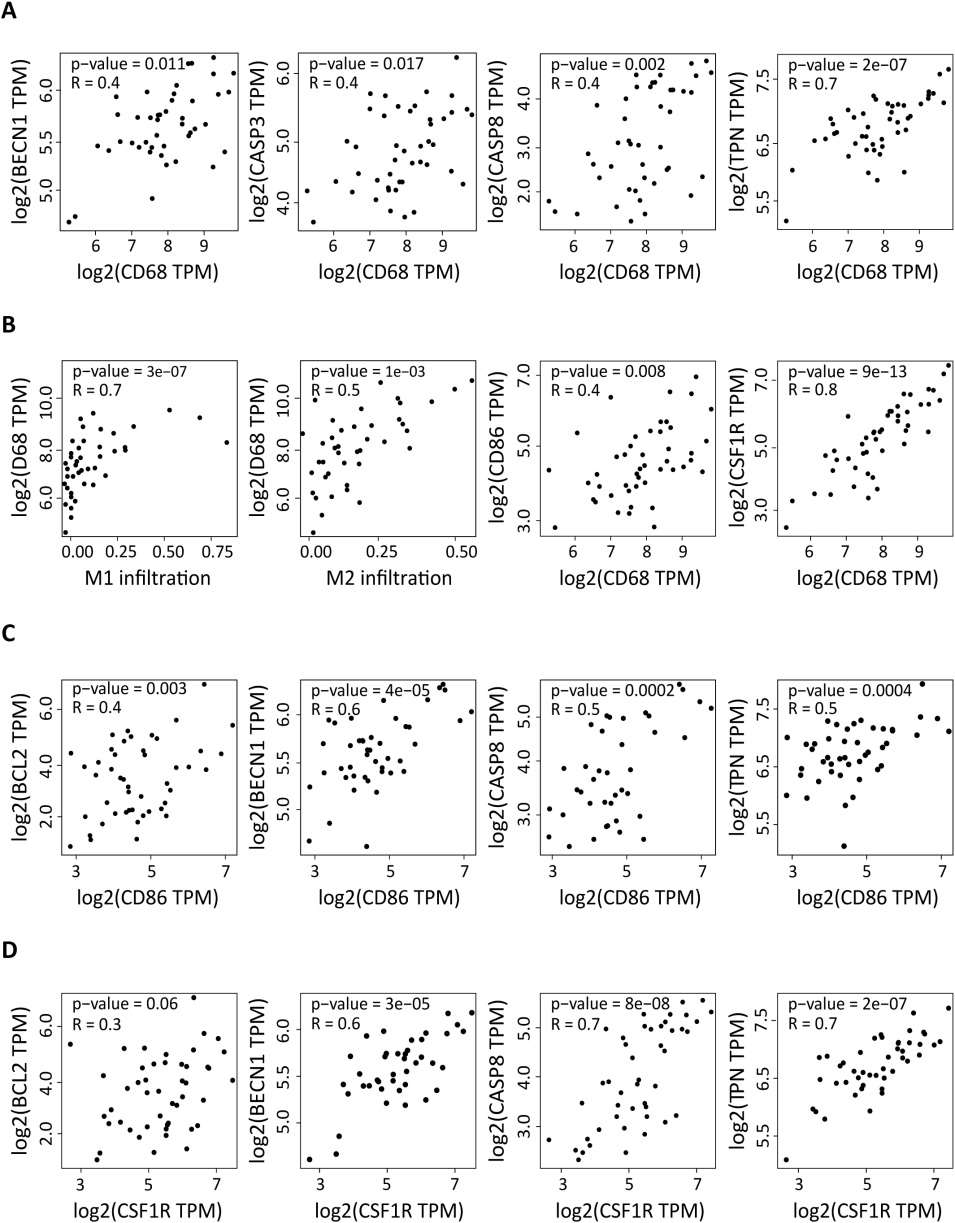
Correlation of the expression of autophagy and apoptosis factors in DLBCL with macrophage infiltration and with the *CD86* and *CSF1R* pro- and anti-inflammatory markers. **(A)** Correlation between the expression levels of *CD68* (pan-macrophage marker) and of *BECN1* and *TPN* (autophagy factors) and *CASP3* and *CASP8* (apoptosis components) in 47 DLBCL samples (TCGA database). **(B)** Correlation between M1 and M2 macrophage infiltration and *CD68* expression level, and correlation of the expression levels of *CD68* and *CD86* and *CSF1R*. **(C)** Correlation between the expression levels of *CD86* (pro-inflammatory marker) and of *BECN1* and *TPN* (autophagy factors) and of *BCL2* and *CASP8* (apoptosis components). **(D)** Correlation between the expression levels of *CSF1R* (anti-inflammation marker) and of *BECN1* and *TPN* (autophagy factors) and of *BCL2* and *CASP8* (apoptosis components). Quantitative comparisons and measures of the strength and direction of the relationship between genes were based on the Pearson’s correlation coefficient (r). Y-axis: log2 (TPM), X-axis: log2 (TPM) of the indicated gene expression levels. TPM: transcript count per million reads.

As we previously showed that the DLBCL immune microenvironment is enriched in macrophages, we asked whether the upregulation of autophagy and apoptosis factors in DLBCL was correlated with the presence of M1 and M2 macrophages. First, we evaluated the correlation between the expression levels of *CD68* (a pan-macrophage marker) (31) and *BECN1*, *NADPH*, *PARG*, *TPN* (autophagy genes) and *BCL2*, *CASP3*, *CASP8*, *CASP9* (apoptosis genes). In DLBCL samples, *CD68* expression was positively correlated with the expression of *BECN1* and *TPN* (r = 0.4, p = 0.011; r = 0.7, p = 2e-07) and of *CASP3* and *CASP8* (r = 0.4, p = 0.017; r = 0.4, p = 0.002) (**Figure 2A**). Then, we evaluated in DLBCL, the correlation of *CD68* expression with M1 and M2 macrophage infiltration and with the expression levels of *CD86* (M1 macrophage marker) and *CSF1R* (M2 macrophage marker) (32, 33). As expected, *CD68* expression was positively correlated with M1 and M2 macrophage infiltration (r = 0.7, p = 3e-07; r =0.5, p = 1e-03) and with *CD86* and *CSF1R* expression levels (r = 0.4, p = 0.008; r = 0.8, p = 9e-13) (**Figure 2B**). Lastly, we determined whether *CD86* and *CSF1R* expression levels were correlated with those of *BECN1*, *NADPH*, *PARG*, *TPN* (autophagy genes) and *BCL2*, *CASP3*, *CASP8*, *CASP9* (apoptosis genes). *CD86* expression was positively correlated with the expression level of *BECN1* and *TPN* (r =0.6, p =4e-05; r =0.5, p =0.0004) and also of *BCL2* and *CASP8* (r = 0.4, p = 0.003; r = 0.5, p = 0.0002) (**Figure 2C**). Moreover, *CSF1R* expression was positively correlated with the expression level of *BECN1* and *TPN* (r = 0.6, p = 3e-05; r = 0.7, p = 2e-07) and of *CASP8* (r = 0.7, p = 8e-08), but not of *BCL2* (r = 0.3, p = 0.06) (**Figure 2D**).

Altogether, these results show that in the DLBCL microenvironment, the increased expression of autophagy and apoptosis genes is correlated with macrophage infiltration. In addition, the expression levels of autophagy and apoptosis genes (except for *BCL2*) are also correlated with *CD86* and *CSF1R* expression. However, *BCL2* expression correlates with *CD86*, but not with *CSF1R* expression.

### In the DLBCL microenvironment, the expression levels of autophagy and of apoptosis genes are increased in M0 and M1 macrophages, and are correlated with mammalian target of rapamycin expression

**Figure 3:**
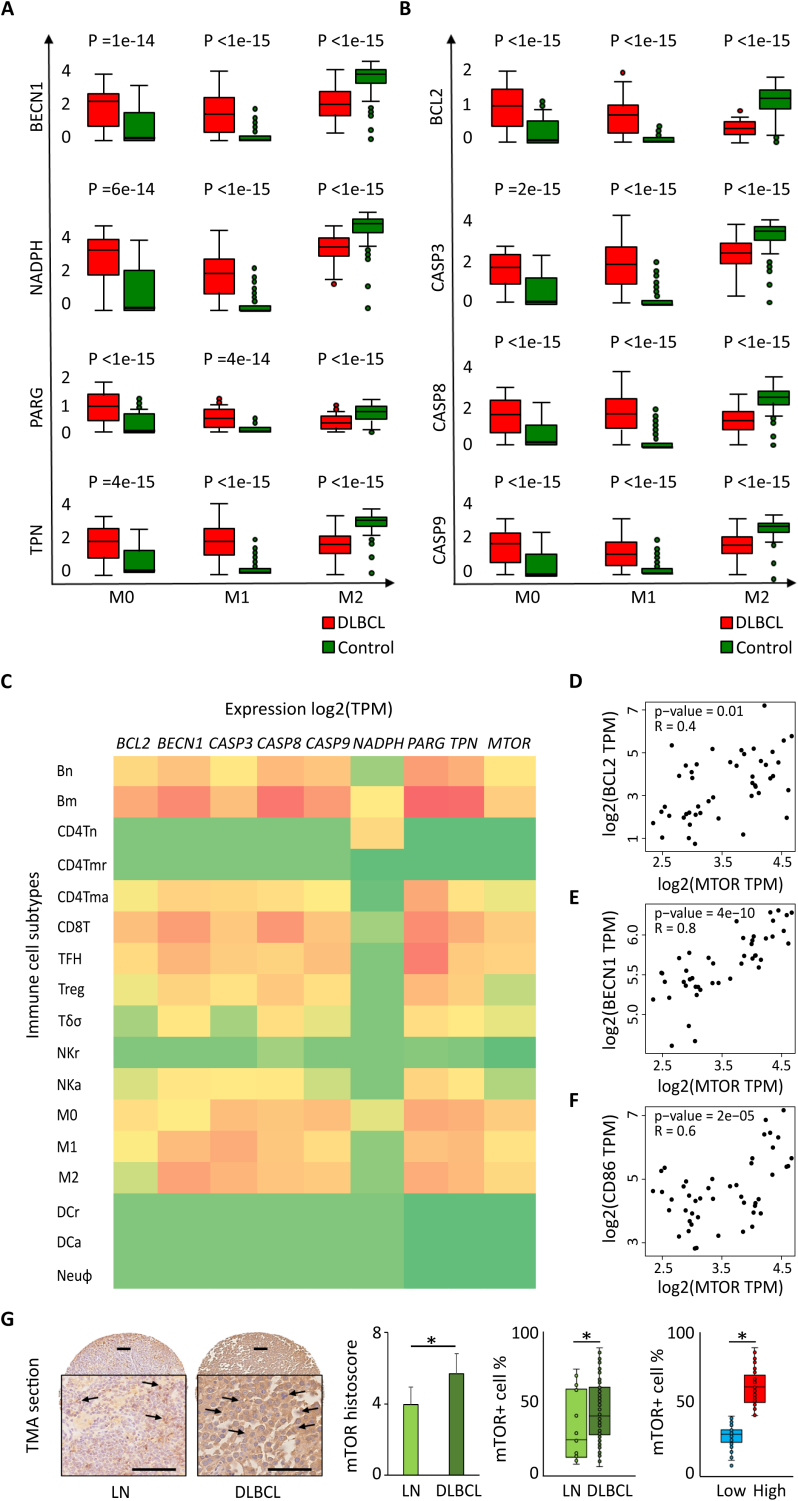
Differential expression of autophagy and apoptotic factors in the DLBCL immune microenvironment and their link with *MTOR* expression. Comparison (one-way ANOVA) of the expression of *BECN1*, *NADPH*, *PARG* and *TPN* (autophagy factors) **(A)** and *BCL2*, *CASP3*, *CASP8* and *CASP9* (apoptosis factors) **(B)** in M0, M1 and M2 macrophages in 47 DLBCL samples (TCGA database) and 337 control samples (normal secondary lymphoid organ tissues; GTEx database). Y-axis: log (TPM + 1) of the gene expression levels. TPM: transcript count per million reads. This figure displays the results of the deconvolution analyses according to the tissue type: DLBCL (red) and control (green). **(C)** Comparison of the log2(TPM) expression levels of *BECN1*, *NADPH*, *PARG*, *TPN* (autophagy factors) and *BCL2*, *CASP3*, *CASP8*, *CASP9* (apoptosis factors) and *MTOR* in different immune cell subtypes of 47 DLBCL samples (TCGA database) and 337 control samples (normal secondary lymphoid organ tissue; GTEx database): naive B cells (Bn), mature B cells (Bm), naive CD4^+^ T cells (CD4Tn), CD4^+^ memory resting T cells (CD4Tmr), CD4^+^ memory activated T cells (CD4Tma), CD8^+^ T cells (CD8T), T follicular helper cells (TFH), regulatory T cells (Treg), gamma delta T cells (Tγδ), resting natural killer cells (NKr), activated natural killer cells (NKa), M0, M1 and M2 macrophages, resting dendritic cells (DCr), activated dendritic cells (DCa), and neutrophils (Neuφ). **(D-F)** Correlation between the expression levels of *MTOR* and *BCL2***(D)**, *BECN1***(E)**, and *CD86***(F)**. Quantitative comparisons and measures of the strength and direction of the relationship between genes were based on the Pearson’s correlation coefficient (r). **(G)** Representative images of mTOR protein expression in normal lymph nodes (LN) and DLBCL. Quantitative analysis of the mTOR histoscore in LN and DLBCL samples (left panel), of the mTOR^+^ cell percentage in LN and DLBCL samples (middle panel), and of mTOR^+^ low and high cell percentage in DLBCL samples (right panel). *p ≤0.05 (two-tailed unpaired Student’s *t*-test).

Next, using deconvolution analysis, we investigated the expression of these autophagy and apoptosis factors specifically in the M0, M1 and M2 macrophage subtypes in DLBCL samples and in non-tumor controls. In line with the previous results (**Figure 2**), we found that key autophagy genes (*BECN1*, *NADPH*, *PARG* and *TPN*) were significantly upregulated in M0 and M1 macrophages in DLBCL compared with control samples (tumor/control fold change: 2.3e+01 to 7.7e+01, p ≤6.0e-14 for M0; 1.3e+02 to 2.1e+03, p ≤4.0e-14 for M1, respectively), but not in M2 macrophages (tumor/control fold change: 2.4e-01 to 6.7e-01, p ≤1.e-15) (**Figure 3A**, **Table 1**). Similarly, key apoptosis genes (*BCL2*, *CASP3*, *CASP8* and *CASP9*) were upregulated in M0 and M1 macrophages in DLBCL compared with control samples (tumor/control fold change: 3.7e+01 to 6.0e+01, p ≤2.0e-15 for M0; 4.5e+02 to 1.4e+03, p ≤1.0e-15 for M1, respectively), but not in M2 macrophages (tumor/control fold change: 3.2e-01 to 5.3e-01, p ≤1.e-15) (**Figure 3B**, **Table 1**). These deconvolution results suggest that in DLBCL, key autophagy and apoptosis factors are mainly upregulated in resting (M0) and pro-inflammatory (M1) macrophages.

**Table 1:** Differentially expressed autophagy and apoptosis factors in the three macrophage subtypes in DLBCL.

	M0			M1			M2			P value
	Tumor	Control	Tumor/Control	Tumor	Control	Tumor/Control	Tumor	Control	Tumor/Control	
**BCL2**	0.835	0.014	6.0e+01	0.451	0.001	4.5e+02	0.312	0.978	3.2e-01	<1.0e-15
**BECN1**	2.468	0.108	2.3e+01	1.716	0.001	1.7e+03	2.370	3.805	6.2e-01	≤1.4e-14
**CASP3**	1.45	0.034	4.3e+01	1.419	0.001	1.4e+03	1.494	2.813	5.3e-01	≤1.3e-15
**CASP8**	1.680	0.045	3.7e+01	0.871	0.001	8.7e+02	1.331	2.612	5.1e-01	≤1.9e-15
**CASP9**	1.017	0.019	5.4e+01	0.694	0.001	6.9e+02	0.843	1.946	4.3e-01	<1.0e-15
**NADPH**	0.383	0.005	7.7e+01	0.132	0.001	1.3e+02	0.137	0.512	2.4e-01	≤4.4e-14
**PARG**	3.025	0.187	1.6e+01	2.149	0.001	2.1e+03	2.971	4.441	6.7e-01	≤5.8e-14
**TPN**	1.999	0.065	3.1e+01	1.753	0.001	1.8e+03	1.759	3.126	5.6e-01	≤4.2e-15

Gene expression [log(TPM + 1)] of autophagy components in M0, M1 and M2 macrophages in DLBCL and control samples: median, tumor/control fold change, and p value. E: Exponential function. p ≤0.05 was considered significant. To simplify the table, only the highest significant p values are shown.

**BCL2**, B-cell lymphoma 2; **BCN1**, beclin-1 gene; **CASP3**, caspase 3; **CASP8**, caspase 8; **CASP9**, caspase 9; **NADPH**, Nicotinamide adenine dinucleotide phosphate; **PARG**, poly (ADP-ribose) glycohydrolase; **TPN**, TAP-associated glycoprotein.

Then, to confirm these results and to compare the expression of autophagy and apoptosis factors in the immune DLBCL microenvironment, we analyzed their expression in several immune cell subtypes: naive B cells (Bn), mature B cells (Bm), CD4^+^ naive T (CD4Tn) cells, CD4^+^ memory resting T (CD4Tmr) cells, CD4^+^ memory activated T (CD4Tma) cells, CD8^+^ T cells, natural killer (NK) cells, T follicular helper (TFH) cells, regulatory T cells (Treg), T gamma delta cells (Tγδ), natural killer resting (NKr) cells, natural killer activated (NKa) cells, M0, M1 and M2 macrophages, resting dendritic cells (DCr), activated dendritic cells (DCa), and neutrophils (Neuφ). Compared with most of the immune cell types under study, autophagy and apoptotic factors were upregulated specifically in M0 and M1 macrophages (except for *NADPH* in M1 and M2 macrophages) (p ≤0.05). We found a similar profile also for CD8^+^ T cells and memory B cells, which should be mostly tumor B cells (**Figure 3C**).

We also compared the expression levels of genes that encode factors implicated in different signaling pathways in different cancer types (from the TCGA database). In NHL samples, we found that *MTOR* was upregulated only in DLBCL samples (**Supplementary Figure S2C**). The same analysis in different immune cell subtypes (listed above) showed that *MTOR* was specifically upregulated in M0 and M1 macrophages and also in B cells and CD8^+^ T cells (p ≤0.05) (**Figure 3C**). Moreover, *MTOR* expression correlated with the expression level of *BECN1* (autophagy), *BCL2* (apoptosis) and *CD86* (M1 macrophage marker) (r = 0.8, p = 4e-10; r =0.4, p = 0.01; r = 0.6, p = 2e-05) (**Figures 3D–F**, respectively). We confirmed that also the expression of mTOR protein was increased in 128 DLBCL samples compared with 20 lymph node controls (TMA sections) (histoscore: 5.7 ± 0.9 vs 4.0 ± 0.8, p =0.05; mTOR^+^ cells: 64.3% ± 14.2 vs 32.6% ± 4.9, p =0.04). In addition, when we evaluated mTOR expression level (low *vs* high; median value used as threshold) in DLBCL samples, we found a higher percentage of mTOR^high^ cells than mTOR^low^ cells (67.3% ± 9.0 *vs* 27.6% ± 8.2, p =2e-03) (**Figure 3G**).

We also investigated whether the expression levels of some of the genes of interest (*BCL2, BECN1, CD86, CSF1R* and *MTOR*) varied in function of the DLBCL stage (I, II, II and IV), but we did not find any significant difference (**Supplementary Figure S3**).

Then, to determine whether the survival of patients with DLBCL (n=48) was influenced by the expression level of these autophagy and apoptosis components, we generated Kaplan Meier survival curves and compared them with the log rank test (survival contribution of each individual gene) and also created survival maps (survival contribution of different genes). We found that the expression level of the tested autophagy and apoptosis factors did not affect survival (**Supplementary Figures S4A-D**). This negative result could be explained by the small size of the cohort (n=48). Therefore, a study in larger cohort of patients with the possibility of functional validation should be carried to confirm/invalidate this negative result.

Altogether, these data suggest that autophagy and apoptosis components as well as *MTOR* are overexpressed in the M0 and M1 macrophage populations of the DLBCL immune microenvironment.

### In the DLBCL microenvironment, the percentage of CD86-positive cells is increased and is accompanied by overexpression of beclin-1 and Bcl-2

**Figure 4:**
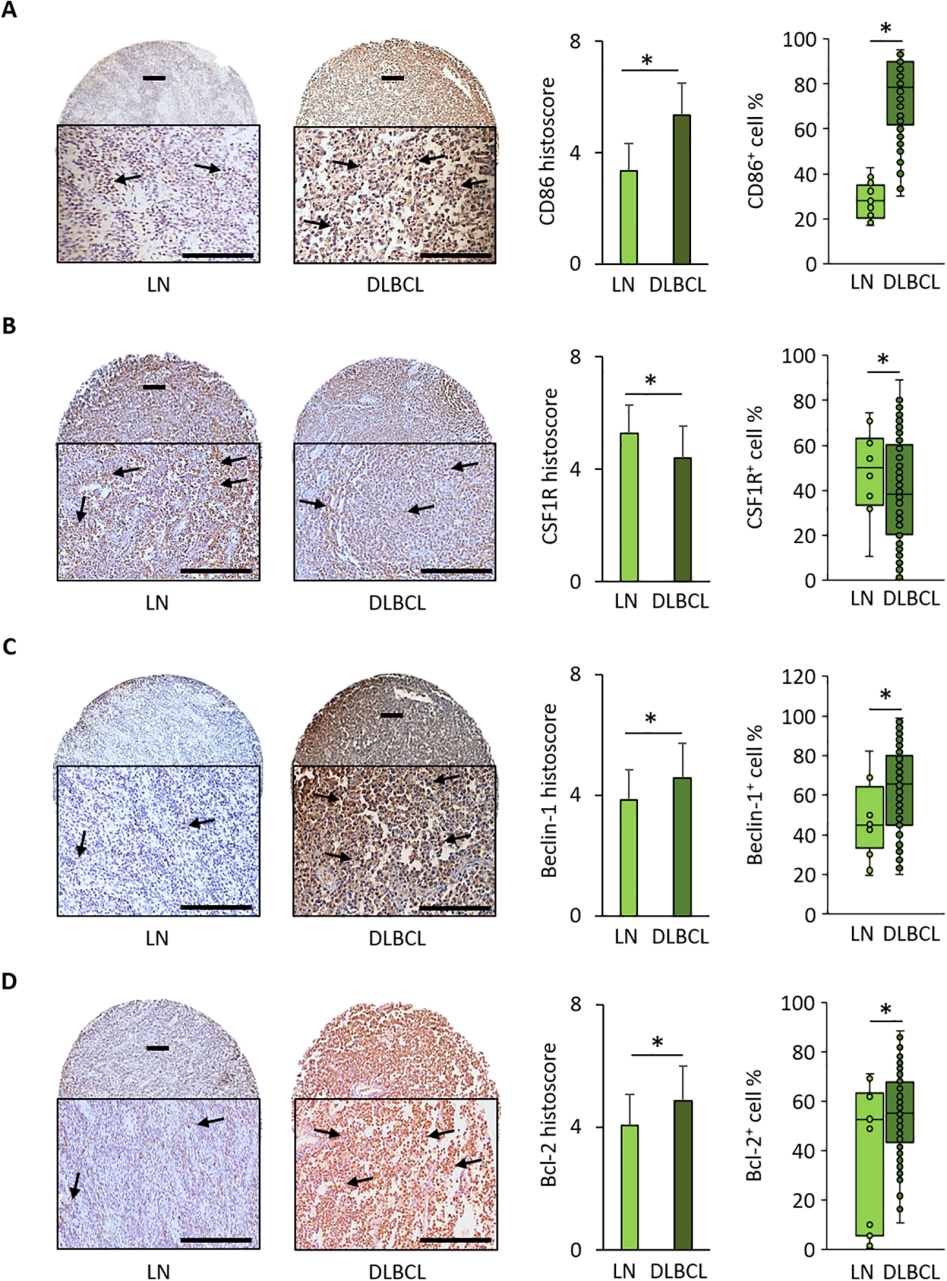
Immunohistochemical analysis of CD86, CSF1-R, Beclin-1, and Bcl-2 expression in 128 DLBCL and 20 normal lymph node samples. **(A)** Representative images of CD86 expression in normal lymph nodes (LN) and DLBCL samples. Quantitative analysis of the CD86 histoscore (left panel) and percentage of CD86^+^ cells (right panel) in DLBCL and LN samples. **(B)** Representative images of CSF1-R expression in LN and DLBCL samples. Quantitative analysis of the CSF1-R histoscore (left panel) and percentage of CSF1-R^+^ cells (right panel) in DLBCL and LN samples. **(C)** Representative images of beclin-1 expression in LN and DLBCL samples. Quantitative analysis of the beclin-1 histoscore (left panel) and percentage of beclin-1^+^ cells (right panel) in DLBCL and LN samples. **(D)** Representative images of Bcl-2 expression in LN and DLBCL samples. Quantitative analysis of the Bcl-2 histoscore (left panel) and percentage of Bcl-2^+^ cells (right panel) in DLBCL and LN samples. Scale bars, 100 µm. *p ≤0.05 (two-tailed unpaired Student’s *t*-test).

To validate some of the *in silico* results, we performed IHC using TMAs (n=128 DLBCL and n=20 normal lymph node samples) (**Supplementary Table S2**). The mean histoscore for CD86 (M1 macrophage marker) was significantly higher in DLBCL samples than in non-tumor controls (5.4 ± 1.1 *vs* 3.4 ± 1.0, p = 9.0e-08) as well as the mean CD86^+^ cell percentage (74.6% ± 17.8 *vs* 27.9% ± 7.8, p = 4.0e-24) (**Figure 4A**).

Conversely, the mean histoscore for CSF1-R (M2 macrophage marker) (4.4 ± 1.3 *vs* 5.3 ± 0.9, p = 0.01) and the mean CSF1-R^+^ cell percentage (40.6% ± 23.3 *vs* 51.8% ± 15.2, p = 0.05) were significantly lower in DLBCL samples than in controls (**Figure 4B**).

The mean beclin-1 and Bcl-2 histoscore values and beclin-1^+^ and Bcl-2^+^ cell percentages were significantly higher in DLBCL than in non-tumor control samples (beclin-1: 4.6 ± 1.3 vs 3.8 ± 1.0, p = 2.0e-02; and 62.9% ± 21.7 *vs* 47% ± 19, p = 4.0e-02, respectively; Bcl-2: 4.9 ± 1.2 *vs* 4.1 ± 2.1, p = 3.0e-01; and 55.8% ± 13.5 *vs* 39.0% ± 7.3, p = 0.05, respectively) (**Figures 4C, D**).

The IHC results obtained in an independent DLBCL cohort confirmed the bioinformatics findings: increased expression of beclin-1 (autophagy factor) and Bcl-2 (anti-apoptosis factor), and higher infiltration of CD86^+^ cells than CSF1-R^+^ cells in the DLBCL microenvironment.

### The pro-inflammatory M1 macrophages are enriched in the peri-tumoral microenvironment and they strongly express Bcl-2 and mTOR

**Figure 5:**
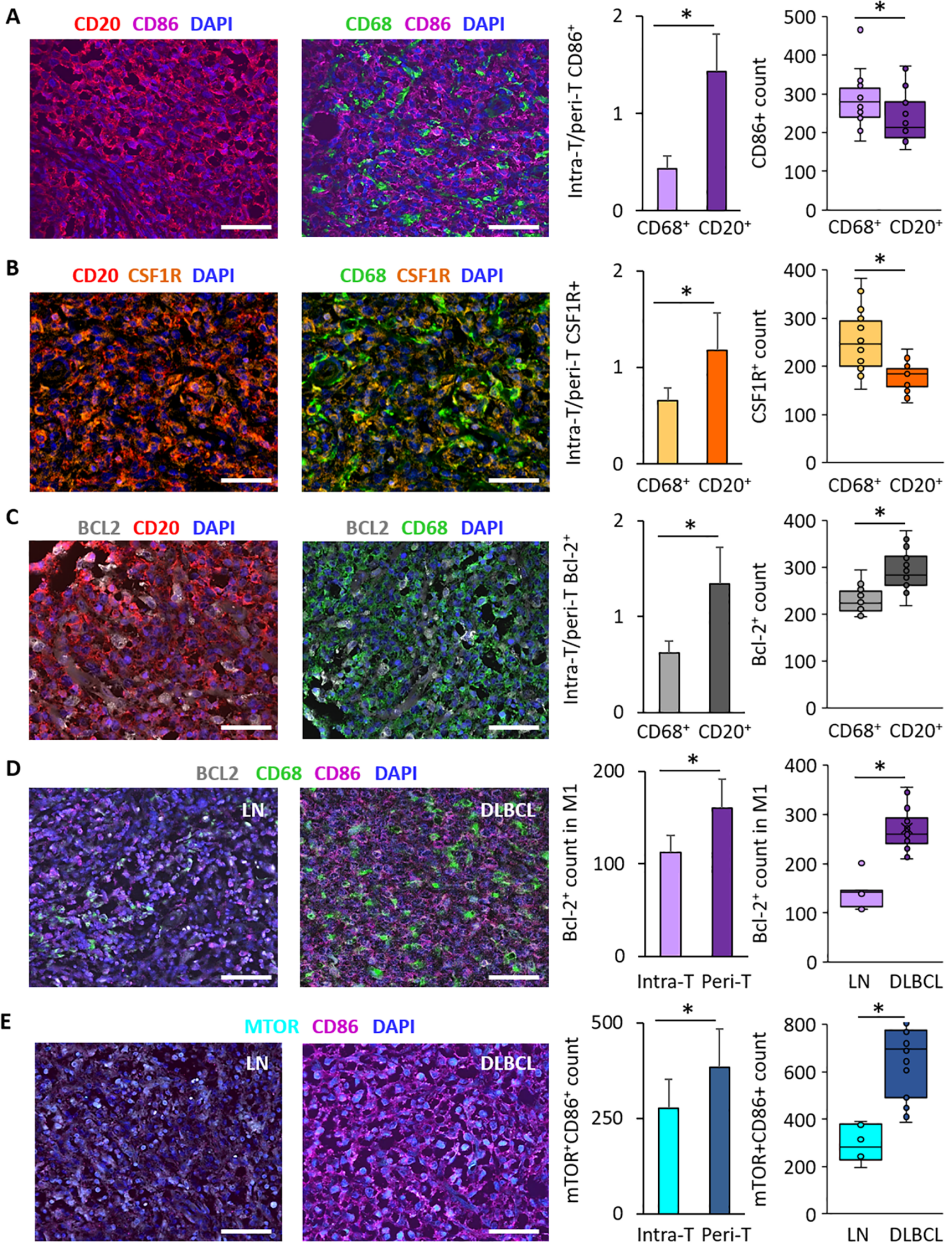
Immunofluorescence analysis of CD86, CSF1-R and Bcl-2 distribution in DLBCL samples and of Bcl-2 and mTOR expression in the DLBCL macrophage microenvironment. **(A)** Representative images of CD86 expression in CD20^+^ tumor cells and CD68^+^ macrophages and its distribution in the DLBCL microenvironment. Quantification of the intra-T/peri-T CD86^+^ cell ratio (left panel) and of CD86^+^ cell numbers (right panel) in the macrophage (CD68^+^) and DLBCL (CD20^+^) compartments. **(B)** Representative images of CSF1-R expression in CD20^+^ tumor cells and CD68^+^ macrophages and its distribution in the DLBCL microenvironment. Quantification of the intra-T/peri-T CSF1-R^+^ cell ratio (left panel) and of the CSF1-R^+^ cell numbers (right panel) in the macrophage (CD68^+^) and DLBCL (CD20^+^) compartments. **(C)** Representative images of Bcl-2 expression in CD20^+^ tumor cells and CD68^+^ macrophages and its distribution in the DLBCL microenvironment. Quantification of the intra-T/peri-T Bcl-2^+^ cell ratio (left panel) and of the Bcl-2^+^ cell numbers (right panel) in the macrophage (CD68^+^) and DLBCL (CD20^+^) compartments. **(D)** Representative images of Bcl-2 expression in M1 macrophages in non-tumor lymph node (LN) controls (left) and DLBCL samples (right). Quantification of Bcl-2^+^ cells in M1 macrophages in the intra- and peri-T areas (left panel) and in LN vs DLBCL samples (right panel). **(E)** Representative images of mTOR expression in CD86^+^ cells in LN (left) and DLBCL samples (right). Quantification of mTOR^+^/CD86^+^ cells in the intra-T and peri-T areas (left panel) and in LN vs DLBCL samples (right panel). Scale bars, 100 µm. *p ≤0.05 (two-tailed unpaired Student’s *t*-test).

To investigate the intra-T and peri-T topography and the link of macrophages (CD68^+^) and tumor B cells (CD20^+^) with CD86 and CSF1-R expression, we performed IF staining of DLBCL and non-tumor control samples in the same TMAs. We found that the intra-T/peri-T ratio of CD86^+^ cells was significantly lower in CD68^+^ (pan-macrophage marker) cells than CD20^+^ tumor B cells (0.4 ± 0.1 *vs* 1.4 ± 0.4, p = 3e-13) (**Figure 5A**). In agreement, CD86^+^ cell count was significantly higher in the macrophage (CD68^+^) than in the tumor cell (CD20^+^) compartment (282.0 ± 63.4 *vs* 238.1 ± 64.3, p = 0.02) (**Figure 5A**).

Similarly, the intra-T/peri-T CSF1-R^+^ cell ratio was significantly lower in the CD68^+^ macrophage than CD20^+^ tumor cell compartment (0.7 ± 0.1 *vs* 1.2 ± 0.3, p = 4e-08) (**Figure 5B**) and CSF1-R^+^ cell count was higher in the macrophage than tumor cell compartment (252.2 ± 61.3 *vs* 177.6 ± 28.7, p = 8e-06) (**Figure 5B**).

Moreover, the intra-T/peri-T Bcl-2^+^ cell ratio was significantly lower in the CD68^+^ than CD20^+^ cell compartment (0.6 ± 0.2 *vs* 1.3 ± 0.3, p = 1e-13) (**Figure 5C**) and Bcl-2^+^ cell count was lower in the macrophage than tumor cell compartment (292.9 ± 40.7 *vs* 228.7 ± 26.5, p = 4e-07) (**Figure 5C**).

Bcl-2 expression was significantly lower in the CD68^+^ macrophage compartment compared with the CD20^+^ tumor cell compartment, but was correlated with M1 (CD86^+^) macrophage infiltration. Therefore, we investigated Bcl-2 expression in CD68^+^CD86^+^ M1 macrophages in DLBCL samples (intra-T *vs* peri-T) and in DLBCL *vs* non-tumor control lymph node samples. In line with the bioinformatics results, Bcl-2 expression in M1 macrophages was significantly lower in the intra-T than peri-T area (112.2 ± 18.9 *vs* 160.3 ± 31.1, p = 4e-07) (**Figure 5D**). In addition, the number of Bcl-2^+^ M1 macrophages was higher in DLBCL samples than controls (272.5 ± 42.6 *vs* 138.5 ± 30.0, p = 7e-09) (**Figure 5D**).

Lastly, as mTOR was overexpressed in DLBCL compared with control samples (**Figure 3G**), we investigated its expression in CD86^+^ cells in DLBCL samples (intra-T *vs* peri-T) and in DLBCL *vs* non-tumor control (lymph nodes) samples. In line with the *in silico* results, the number of mTOR^+^ CD86^+^ cells was significantly lower in the intra-T than peri-T area (276.2 ± 75.0 *vs* 383.8 ± 100.4, p = 4e-04) and was higher in DLBCL than control samples (660.1 ± 154.7 *vs* 2 94.5 ± 78.3, p = 8e-06) (**Figure 5E**).

These IF results validated and complemented our *in silico* and IHC findings. They suggest that tumor-infiltrating M1 (CD86^+^) and M2 (CSF1-R^+^) macrophages have a peri-T location and that more macrophages (CD68^+^) than DLBCL cells (CD20^+^) express these two markers. In addition, Bcl-2 expression was higher in CD20^+^ tumor cells (a lymphoma feature) than in CD68^+^ macrophages. Specifically, in the macrophage compartment, Bcl-2^+^ macrophages were located in the peri-T area and predominantly belonged to the M1 subpopulation. Lastly, mTOR was mainly expressed in CD86^+^ cells in the peri-T area and its expression level was higher in DLBCL than control samples.

## Discussion

DLBCL is characterized by an important immune microenvironment that influences its prognosis and outcome (20, 34–36). This aggressive blood cancer needs a substantial amount of energy to highjack the immune cell microenvironment and deregulate vital physiological processes (inflammation, autophagy and apoptosis) in favor of tumor growth and progression. Physiologically, autophagy is an important source of energy for basic cellular processes and for the activation/regulation of the innate immune response (37). In tumor cells, autophagy deregulation could hinder the antitumor immune response induction, due to insufficient ATP release to attract immune cells (38). One study reported an autophagy signature that is related to DLBCL resistance to drugs (39). Hence, the relationship between autophagy and immune cell response is crucial particularly in cancer.

This study is the last part of a project that exploited publicly available datasets that contain 48 DLBCL tumors and 337 non-tumor control samples and then used a substantial independent cohort of 128 DLBCL tumors and 38 control samples (including 20 lymph nodes) in TMAs to validate the *in silico* results by IHC and IF. The first part of this project highlighted a significant and high enrichment in macrophages, specifically pro-inflammatory M1 macrophages, and a strong inflammatory signature in the DLBCL microenvironment (20). In the second part of the project, we hypothesized that DLBCL would require a high amount of energy to maintain this strong inflammatory signature. By investigating the expression of several metabolic components, we found that the metabolic regulator SIRT1 is upregulated in the DLBCL microenvironment. Moreover, SIRT1 expression was correlated with M1 pro-inflammatory macrophages and was linked to important metabolic pathways, such as autophagy. This suggested a specific relation between metabolic targets and inflammation in the DLBCL (manuscript under revision).

Here, in the last part of this project, we investigated the autophagy gene expression profile and its potential link with inflammation in the DLBCL microenvironment and in non-tumor controls. As expected, autophagy genes were upregulated in the DLBCL microenvironment, including *BECN1* that is involved in autophagy initiation (17). Moreover, *BCL2* (an anti-apoptotic factor overexpressed and often mutated in DLBCL) (40) was also upregulated in our DLBCL cohort and its expression was correlated with that of *CASP3* and *CASP8* (two apoptosis regulators). *BCL2* expression was also positively correlated with that of *BECN1*, suggesting a strong relationship between these factors in DLBCL. This is in line with what described in a physiological context (17, 41) where their interaction is crucial for autophagy regulation and cellular homeostasis. Moreover, *BECN1* expression was correlated specifically with the CD68^+^ macrophage compartment and with both M1 (CD86^+^) and M2 (CSF1-R^+^) macrophage subtypes. Conversely, *BCL2* expression was only positively correlated with M1 pro-inflammatory macrophages. When we verified *BECN1* and *BCL2* expression levels in the M0, M1 and M2 macrophage subtypes in DLBCL and non-tumor control samples, we found that they were overexpressed in M0 and M1 but not in M2 macrophages. In addition, the comparison with other immune cell types showed an overall upregulation of the studied autophagy and apoptosis components only in the rich DLBCL macrophage compartment (and also in tumor B cells and CD8^+^ T cells). Altogether, this suggests a link between autophagy and inflammation. Moreover, in DLBCL, the autophagy machinery might decrease the sensitivity of macrophages to death by apoptosis (42–44), specifically in M1 pro-inflammatory macrophages, to support their anti-tumor inflammatory response. The validation of these results by IHC and IF in a substantial independent cohort of 128 DLBCL and 38 non-tumor control samples on TMAs allowed confirming the significantly higher proportion of pro-inflammatory (CD86^+^) cells than suppressive (CSF1-R^+^) cells in DLBCL compared with controls, and also the overexpression of beclin-1 and Bcl-2 in DLBCL. Moreover, using IF, we determined the specific topography of CD20^+^ tumor cells (intra-T area) and of CD68^+^CD86^+^ M1 and CD68^+^CSF1-R^+^ M2 macrophages (peri-T area) in DLBCL samples. We also showed that Bcl-2 is overexpressed in tumor B cells and in the peri-T M1 macrophage compartment.

Lastly, we previously reported that the mTOR signaling pathway, a central regulator of metabolism (45), is enriched in the DLBCL microenvironment (manuscript under revision). Here, we found that *MTOR* expression correlates with CD68^+^ macrophage infiltration and with both *BECN1* and *BCL2* expression. In addition, mTOR expression was higher in DLBCL than non-tumor control samples, and in tumors, mTOR expression was higher in CD86^+^ cells in the peri-T area. Based on the literature and our previous results, we hypothesize that overexpression of mTOR in tumor cells and in M1 macrophages could be an adaptation induced by tumor cells (20, 46, 47) to limit inflammation and to negatively regulate autophagy, which is implicated in generating enough energy to activate these innate immune cells and to support the inflammatory reaction. Furthermore, this extra energy could be exploited by tumor cells for their own metabolism and growth. It would be important to perform functional studies and phosphorylation-specific assays to verify/confirm whether mTOR overexpression pattern reflects its activation status.

**Figure 6:**
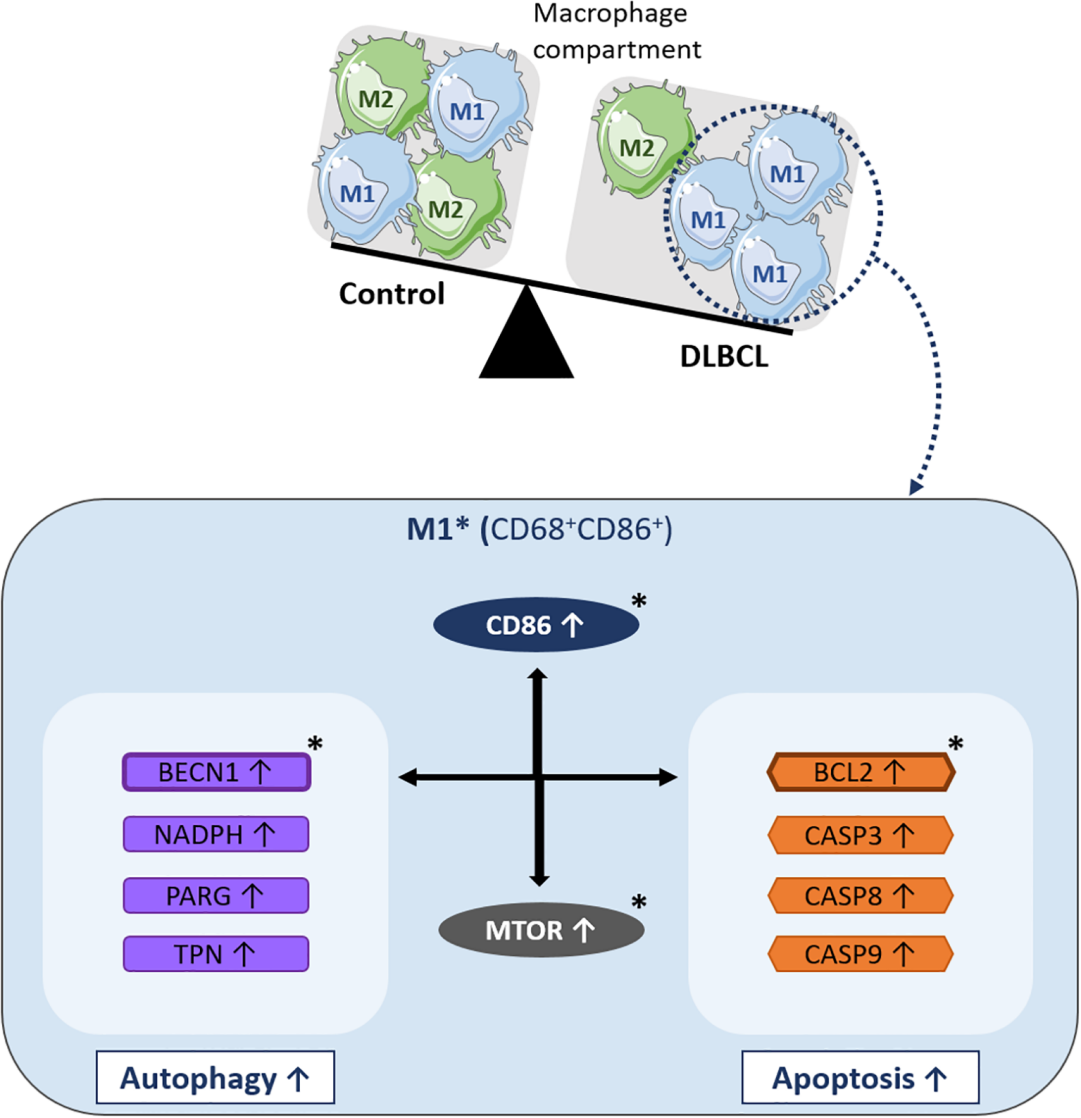
Schematic model of the molecular events underlying *BCL2*-related autophagy gene upregulation in M1 macrophages of the DLBCL microenvironment. In the macrophage compartment, the proportion of pro-inflammatory M1 macrophages is increased in DLBCL compared with non-tumor controls. Many key autophagy (*BECN1*, *NADPH*, *PARG*, and *TPN*) and apoptosis genes (*BCL2*, *CASP3*, *CASP8*, and *CASP9*) are upregulated in M1 (CD68^+^ CD86^+^) macrophages in the DLBCL microenvironment. Among these autophagy genes, *BECN1* expression is correlated with that of most of the autophagy and apoptosis genes analyzed in this study. Among the apoptosis genes, *BCL2* expression is correlated with that of several autophagy and apoptosis factors. Particularly, *BECN1* and *BCL2* expression levels are strongly correlated and both are correlated with *MTOR* expression (a key factor involved in important biological processes, such as inflammation and autophagy). *BCL2*, *BECN1* and *MTOR* expression levels are also correlated with the expression of *CD86*, a main pro-inflammatory factor and one of the most important markers of M1 macrophages and inflammatory states. *indicates key factors validated at the protein level by immunohistochemistry and/or immunofluorescence analyses.

Altogether, our gene and protein expression results describe for the first time in DLBCL a specific autophagy gene profile in the M1 pro-inflammatory macrophage compartment that is positively correlated with upregulation of the *BCL2* anti-apoptotic factor (**Figure 6**).

Zhou et al. developed a new autophagy-related gene signature to predict the prognosis and resistance to treatment in patients with DLBCL (39). They selected 309 autophagy-related genes from the Human Autophagy Database and GenCards database. Their final autophagy signature contained five genes: *TP53INP2*, identified as a risk gene, and *PRKCQ*, *TUSC1*, *PRKAB1* and *HIF1A* as protective genes. Their expression level in patients allows determining their risk score and classifying them in two groups: high-risk (with poorer overall survival) and low-risk (with better overall survival). The authors showed that this gene signature offers a better prognostic stratification compared with classical methods (such as the International Prognostic Index scoring system). In addition, they found higher immune cell infiltration and immune activation in the low-risk group. Unlike in our survival analysis, the autophagy-related gene signature described by Zhou at al. can predict patient survival (likely due to the different size of the two patient cohorts: n=48 in our study *vs* n=412 in the study by Zhou et al). However, they did not investigate the immune cell type in which autophagy gene expression was upregulated and the correlation with other key physiological process, such inflammation and apoptosis. Therefore, both studies bring independent and additional findings on the importance of autophagy gene expression in DLBCL.

Although TMAs are one of the most powerful ways to investigate *in situ* protein expression (IHC) and interaction/co-localization (IF), the first limitations of our study is the lack of a substantial cohort of fresh patient samples to confirm our hypotheses at the single-cell and functional level. Indeed, functional studies are now needed to confirm the link between autophagy (beclin-1), apoptosis (Bcl-2) and macrophages as well as functional overexpression assays in polarized macrophages. The second important limitation is the reliance on *in silico* analyses, although we believe that this is a powerful and useful tool that allows the exploitation/interpretation of tremendous data amounts. In future work, we want to understand the multifaceted roles of autophagy in inflammation in the context of DLBC and clarify its involvement in cell death. As autophagy seems to play an important role in this aggressive blood cancer, it would be interesting to test/develop therapeutics to target this process, for instance nano-targeting and/or modulation of key autophagy and apoptosis components (such as beclin-1 and Bcl-2) in M1 macrophages. This approach might open new avenues for DLBCL treatment, while reducing drug toxicity (48–50).

## Data Availability

The original contributions presented in the study are included in the article/**Supplementary Material**. Further inquiries can be directed to the corresponding author.

## Supplementary material

Supplementary Figure 1 Circular charts summarizing the patients’ characteristics (TGCA samples): survival **(A)**, tumor type **(B)**, age range **(C)**, sex **(D)**, and treatments **(E)**.

Supplementary Figure 2 Expression levels of *BCL2***(A)**, *BECN1***(B)** and *MTOR***(C)** (from the TCGA database) in different cancer types (ACC: Adrenocortical carcinoma, BLCA, Bladder Urothelial Carcinoma; BRCA, Breast invasive carcinoma; CESC, Cervical squamous cell carcinoma and endocervical adenocarcinoma; CHOL, Cholangiocarcinoma; COAD, Colon adenocarcinoma; DLBC, Diffuse Large B-cell Lymphoma; ESCA, Esophageal carcinoma; GBM, Glioblastoma multiforme; HNSC, Head and neck squamous cell carcinoma; KICH, Chromophobe renal cell carcinoma; KIRC, Clear cell renal cell carcinoma; KIRP, Papillary renal cell carcinoma; LAML, Acute Myeloid Leukemia; LGG, Lower Grade Glioma; LIHC, Hepatocellular carcinoma; LUAD, Lung adenocarcinoma; LUSC, Lung squamous cell carcinoma; MESO; Mesothelioma; OV, Ovarian serous cystadenocarcinoma; PAAD, Pancreatic adenocarcinoma; PCPG, Pheochromocytoma and paraganglioma; PRAD, Prostate adenocarcinoma; READ, Rectum adenocarcinoma; SARC; Sarcoma; SKCM, Cutaneous melanoma; STAD, Stomach adenocarcinoma; TGCT, Testicular germ cell tumors; THCA, Thyroid carcinoma; THYM; Thymoma; UCEC, Uterine corpus endometrial carcinoma; UCS, Uterine carcinosarcoma; UVM, Uveal melanoma). p ≤0.05 was considered significant.

Supplementary Figure 3 Violin plots displaying the expression profiles of *BCL2***(A)**, *BECN1***(B)**, *CD86***(C)**, *CSF1R***(D)** and *MTOR***(E)** in the 48 DLBCL samples (TCGA dataset) classified in function of their stage. p ≤0.05 was considered significant.

Supplementary Figure 4 Kaplan-Meier survival curves based on the expression levels (high vs low) of *BECN1, NADPH, PARG* and *TPN* (autophagy genes) **(A)** and of *BCL2, CASP3, CASP8* and *CASP9* (apoptosis genes) **(B)** in 48 DLBCL samples (TCGA dataset) (**Supplementary Table S4**). **(C)** Survival maps based on the comparison of the survival contribution of *BECN1, NADPH, PARG* and *TPN*, estimated using the Mantel–Cox test. **(D)**. Survival maps based on the comparison of the survival contribution of *BCL2, CASP3, CASP8* and *CASP9*, estimated using the Mantel–Cox test.

Supplementary Table 1 List of the characteristics of patients with DLBCL from the TCGA dataset.

Supplementary Table 2 List of the characteristics of the patients with DLBCL included in the TMAs used for the IHC and IF analyses.

Supplementary Table 3 List of the characteristics of the control samples included in the TMAs used for the IHC and IF analyses.

Supplementary Table 4 List of the differentially expressed autophagy- and apoptosis-related genes in DLBCL and control samples with their respective fold change and p values. P ≤0.05 was considered significant.
