# Supplementary figures and images for: In the diffuse large B-cell lymphoma microenvironment, autophagy genes are upregulated in pro-inflammatory macrophages and linked to BCL2 overexpression
Source: Front Immunol. 2025 Dec 3;16:1676563. doi: 10.3389/fimmu.2025.1676563 (PMC12708328; doi:10.3389/fimmu.2025.1676563)

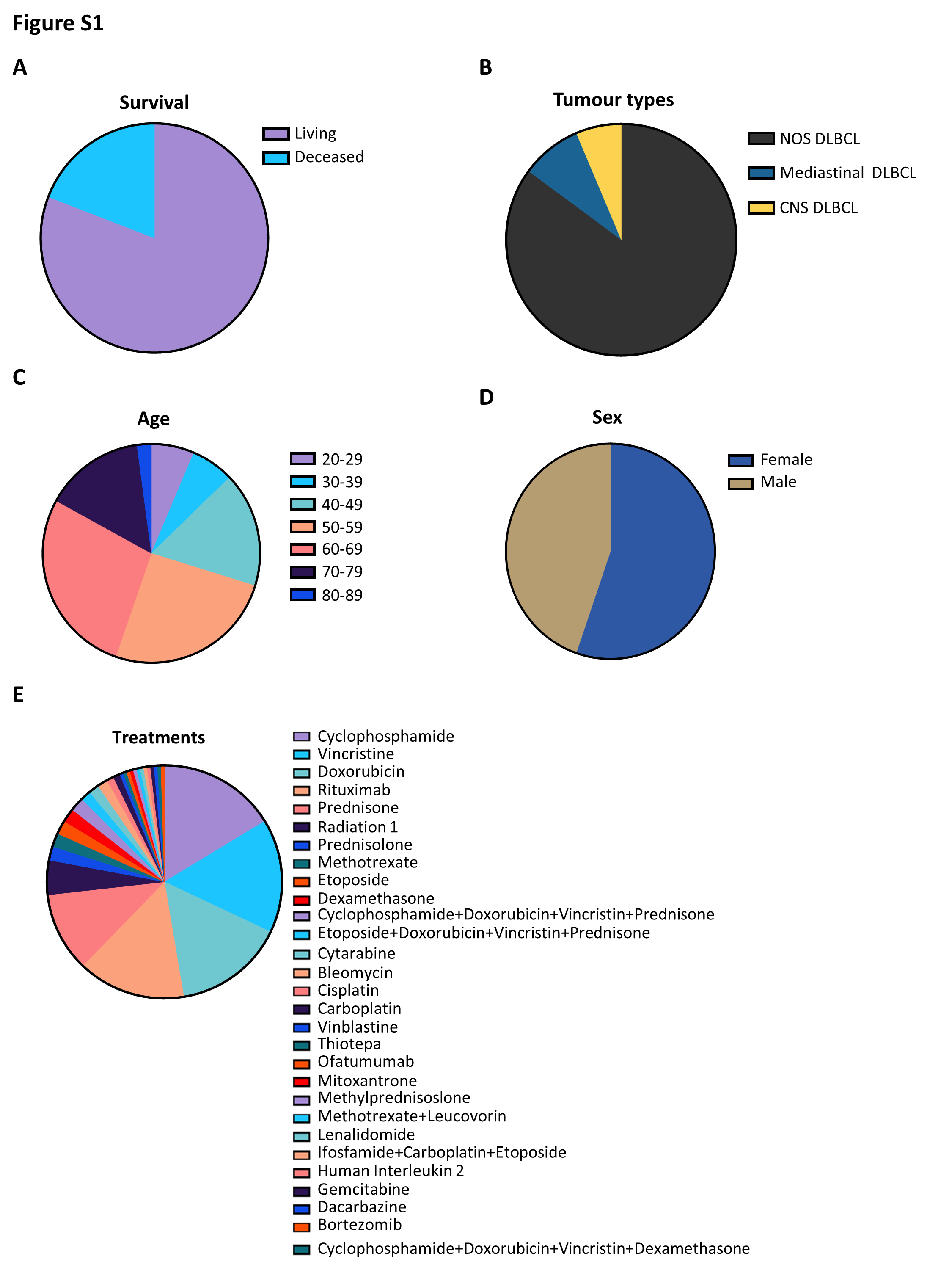

Supplement: Supplementary Figure 1 — Circular charts summarizing the patients’ characteristics (TGCA samples): survival (A), tumor type (B), age range (C), sex (D), and treatments (E). [file Image1.tif]

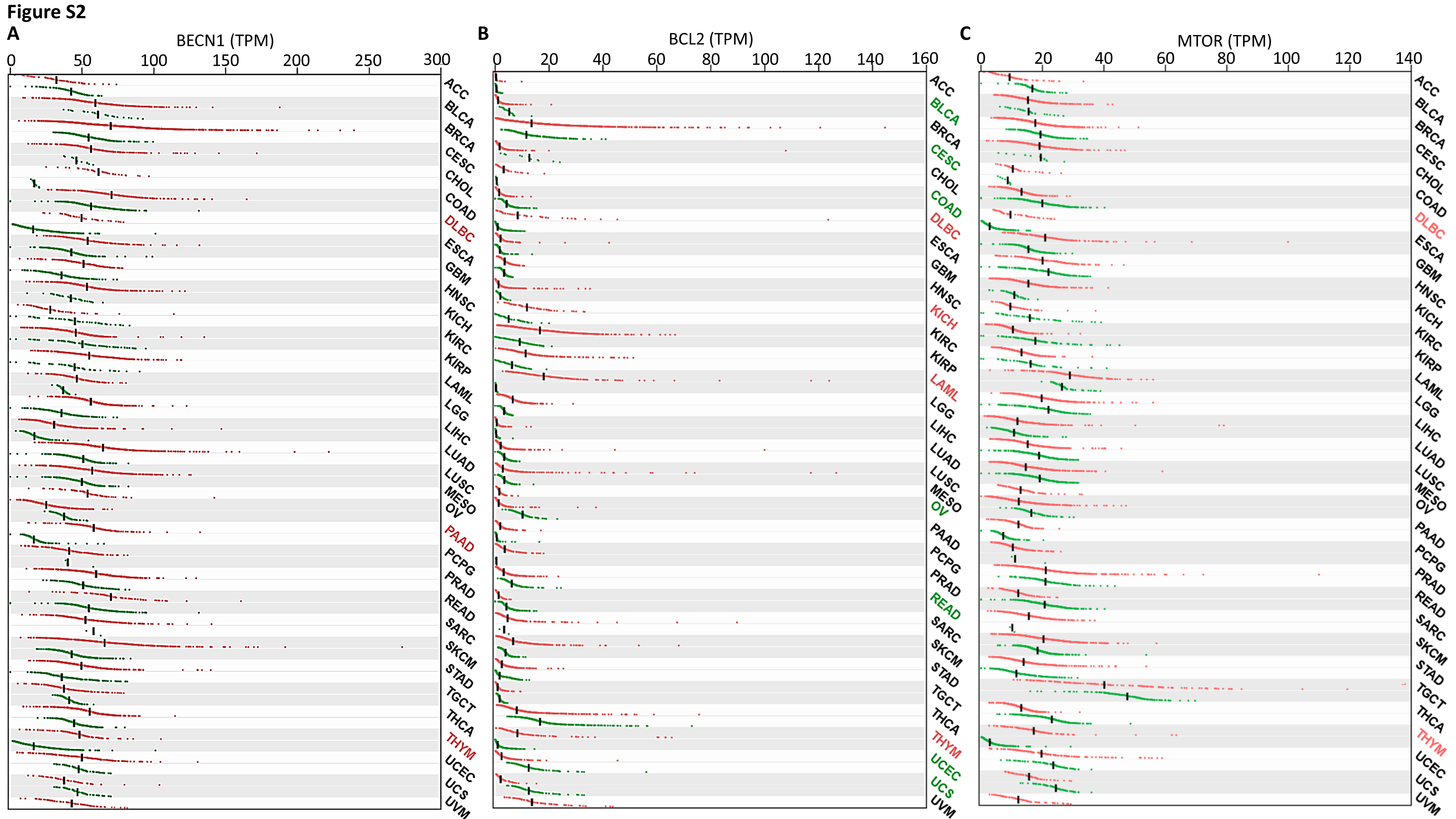

Supplement: Supplementary Figure 2 — Expression levels of BCL2(A), BECN1(B) and MTOR(C) (from the TCGA database) in different cancer types (ACC: Adrenocortical carcinoma, BLCA, Bladder Urothelial Carcinoma; BRCA, Breast invasive carcinoma; CESC, Cervical squamous cell carcinoma and endocervical adenocarcinoma; CHOL, Cholangiocarcinoma; COAD, Colon adenocarcinoma; DLBC, Diffuse Large B-cell Lymphoma; ESCA, Esophageal carcinoma; GBM, Glioblastoma multiforme; HNSC, Head and neck squamous cell carcinoma; KICH, Chromophobe renal cell carcinoma; KIRC, Clear cell renal cell carcinoma; KIRP, Papillary renal cell carcinoma; LAML, Acute Myeloid Leukemia; LGG, Lower Grade Glioma; LIHC, Hepatocellular carcinoma; LUAD, Lung adenocarcinoma; LUSC, Lung squamous cell carcinoma; MESO; Mesothelioma; OV, Ovarian serous cystadenocarcinoma; PAAD, Pancreatic adenocarcinoma; PCPG, Pheochromocytoma and paraganglioma; PRAD, Prostate adenocarcinoma; READ, Rectum adenocarcinoma; SARC; Sarcoma; SKCM, Cutaneous melanoma; STAD, Stomach adenocarcinoma; TGCT, Testicular germ cell tumors; THCA, Thyroid carcinoma; THYM; Thymoma; UCEC, Uterine corpus endometrial carcinoma; UCS, Uterine carcinosarcoma; UVM, Uveal melanoma). p ≤0.05 was considered significant. [file Image2.tif]

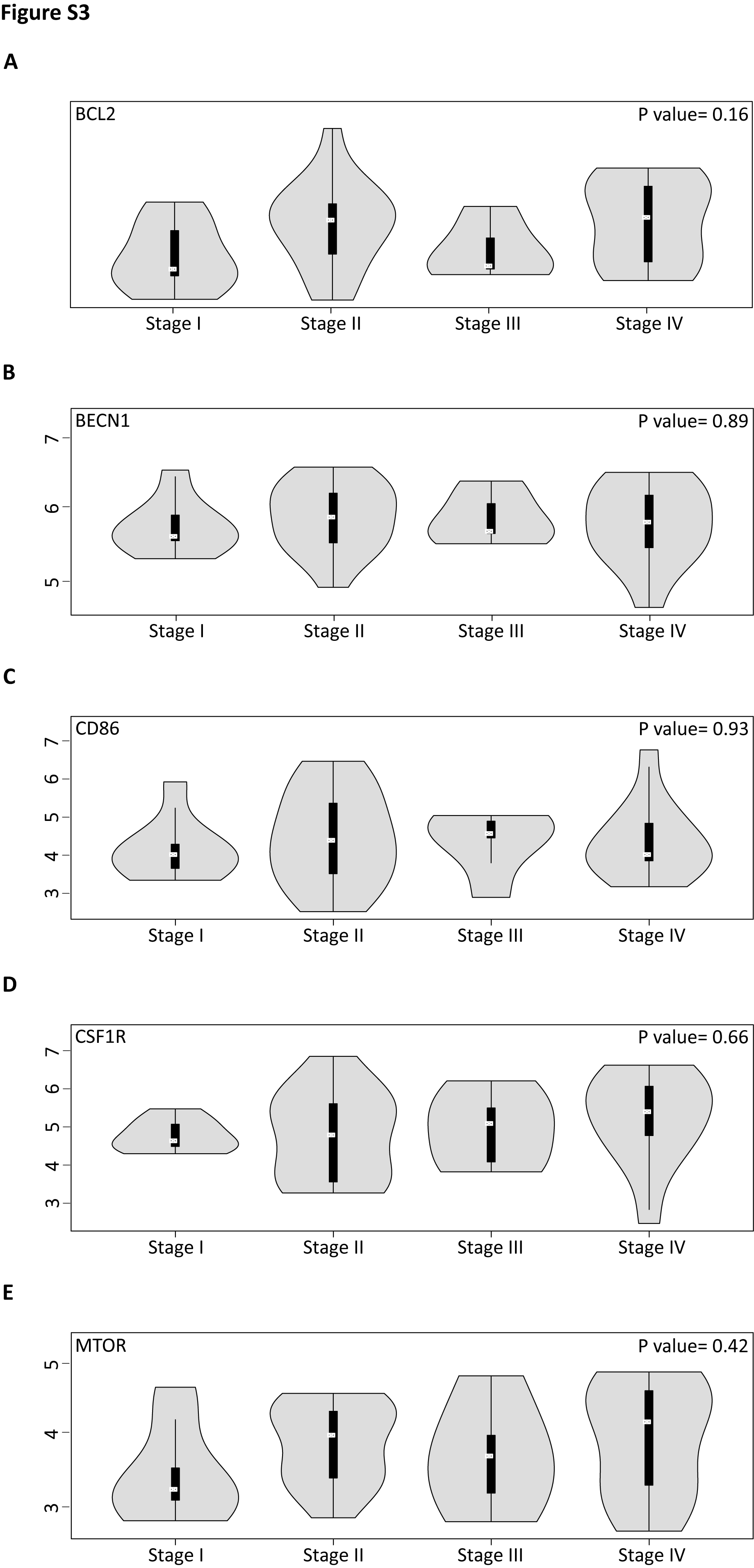

Supplement: Supplementary Figure 3 — Violin plots displaying the expression profiles of BCL2(A), BECN1(B), CD86(C), CSF1R(D) and MTOR(E) in the 48 DLBCL samples (TCGA dataset) classified in function of their stage. p ≤0.05 was considered significant. [file Image3.tif]

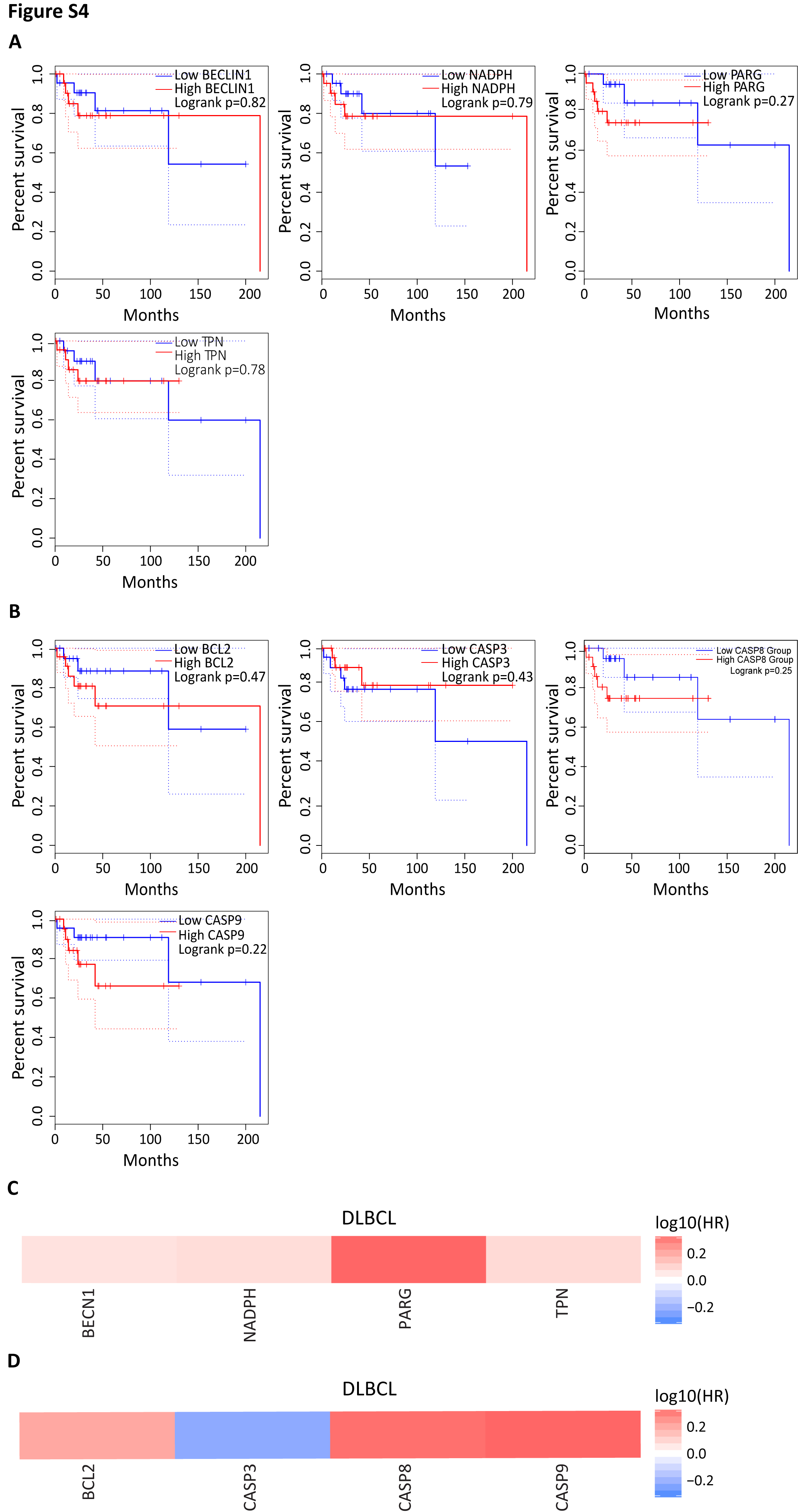

Supplement: Supplementary Figure 4 — Kaplan-Meier survival curves based on the expression levels (high vs low) of BECN1, NADPH, PARG and TPN (autophagy genes) (A) and of BCL2, CASP3, CASP8 and CASP9 (apoptosis genes) (B) in 48 DLBCL samples (TCGA dataset) (Supplementary Table S4). (C) Survival maps based on the comparison of the survival contribution of BECN1, NADPH, PARG and TPN, estimated using the Mantel–Cox test. (D). Survival maps based on the comparison of the survival contribution of BCL2, CASP3, CASP8 and CASP9, estimated using the Mantel–Cox test. [file Image4.tif]
