# Supplementary material for: In the diffuse large B-cell lymphoma microenvironment, autophagy genes are upregulated in pro-inflammatory macrophages and linked to BCL2 overexpression
Source: Front Immunol. 2025 Dec 3;16:1676563. doi: 10.3389/fimmu.2025.1676563 (PMC12708328; doi:10.3389/fimmu.2025.1676563)
Supplement: Supplementary Table 4 — List of the differentially expressed autophagy- and apoptosis-related genes in DLBCL and control samples with their respective fold change and p values. P ≤0.05 was considered significant. [file Table4.docx]

**Table S4.**

| **Gene Symbol** | **Gene ID** | **Median (Tumor)** | **Median (Normal)** | **Log2**  **(Fold Change)** | **adjp** |
| --- | --- | --- | --- | --- | --- |
| BCL2 | ENSG00000171791.11 | 8.400 | 0.950 | 2.269 | 5.14e-28 |
| BECN1 | ENSG00000126581.12 | 49.652 | 15.930 | 1.581 | 1.13e-47 |
| CASP3 | ENSG00000164305.17 | 24.501 | 3.440 | 2.522 | 3.51e-30 |
| CASP8 | ENSG00000064012.21 | 9.280 | 26.891 | -1.440 | 4.91e-7 |
| CASP9 | ENSG00000132906.17 | 10.890 | 4.160 | 1.204 | 1.64e-14 |
| NADPH | ENSG00000104325.6 | 72.448 | 32.039 | 1.153 | 1.21e-5 |
| PARG | ENSG00000227345.8 | 6.760 | 1.090 | 1.892 | 1.89e-26 |
| TPN | ENSG00000231925.11 | 117.538 | 54.258 | 1.101 | 7.59e-6 |
